# The Hospital Debates

**Published:** 1921-07-30

**Authors:** 


### THE HOSPITAL DEBATES.

As in the previous week, two related events have again occurred of importance to the hospital situation. In the House of Lords, on July 20, Viscount Burnham asked the Government what further steps it proposed to take to help the voluntary hospitals, particularly in relation to Poor-law infirmaries, and an interesting debate followed, in which Lord Dawson of Penn took a helpful part. On the same day the British Hospitals Association gave a luncheon to Lord Cave and the members of the Voluntary Hospitals' Committee, Sir Arthur Stanley, President of the Association, presiding. Lord Cave spoke on both occasions, and this emphasises the link between the two. At the luncheon Sir Arthur Stanley said that the crisis was precipitated because the hospitals were facing five or six years of arrears at the moment when prices were at their highest, but he believed that "the upward curve of receipts" would be very remarkable this year. The problem therefore was one of immediate needs. To have cut the Government grant down to £500,000 was to have erred on the side of over-economy, but if even this were distributed in the quickest and wisest way it would be of eminent advantage. Mr. Wade-Deacon took a larger view perhaps when he said that the hospital not in need to-day might be in need shortly, and asked frankly if it were worth while to form innumerable committees when the sum to be distributed was so small. Without money to distribute the hospitals would never get into line. Lord Hambleden, on not dissimilar lines, emphasised the extreme urgency of the need in London, and remarked that if the grant was not distributed quickly even more beds might have to be closed. There was at present too much competition and too little co-operation among hospitals. Lord Cave, in reply, gave what comfort he could by remarking that, except for the amount of the grant, the Government had practically accepted the Committee's recommendations, adding, in a curious metaphor, the hint that the hospitals might look out for "a remount" in a year's time. He then turned to employers and, remarking on the willingness of the masses to contribute, urged manufacturers to transfer the amount of their excess profits duties, now about to come to an end, to the hospitals. As the grant was so small, Lord Cave hoped that it would not be distributed mechanically. Perhaps the League of Mercy would act as a sort of fairy godmother to the local committees in regard to a proportion of the fund. The provinces needed a body similar to King Edward's Hospital Fund for London. Very similar points were made in the debate in the House of Lords on the same afternoon, as will be seen by our summary below.

In sum, the immediate need is to distribute the grant without a moment's delay to those at present most in need of it. This done, two general plans of co-ordination for London and the provinces must be devised : in the matter of beds between the hospitals and the infirmaries; in the matter of finance between the hospitals themselves. Those who form one group for raising income should then see if expenditure cannot be decreased by avoiding overlapping. Those who co-operate to raise income must try to co-operate also in saving expense. This, perhaps, is the second lesson of these discussions.

### The House of Lords and Hospital Finance.

Lord Burnham said that the report of the Cave Committee was necessarily provisional, and dealt, according to the reference, with the present acute position more than with future provision of hospital accommodation. A report of the Chief Medical Officer of the Ministry of Health revealed the fact that in England and Wales there were lost to the nation every year, amongst the insured population alone, the equivalent of the work of 270,000 persons. There was a clear case for asking the Committee to report on two great subjects which were not only ripe for consideration, but were long overdue. One of these was the relation that the system of National Health Insurance bore to the rest of the problem of national health, and particularly to the provision of hospitals, both general and special. The other was the relation of the Poor-law infirmaries to voluntary hospitals.

### The Panel Practitioner and Hospitals.

Lord Burnham did not consider that the Cave Committee had appreciated the degree in which the obligations and burdens of hospitals had been increased by the Insurance Act. No doubt the nation's health had been greatly bettered by the operation of the Act, but hospital treatment was absolutely necessary to obtain those results. The general practitioners had not given all that the Act demanded, or all, perhaps, that they might have given if it had not been for the goodwill and the zealous assistance of the hospital authorities. The consequence was that the Exchequer was getting indirectly the benefit of a vast amount of hospital expenditure. He believed that a clear case existed under the Act for obtaining a contribution out of the surpluses of the approved societies, such as might be possible without an amendment of the law. Further, during the war the vast bulk of the people, and especially those who enlisted in the Forces, came to demand a higher standard of medical treatment, and received it, and they were not willing to revert to the old order, and were not satisfied now merely with the panel doctor, not at his best, but at his worst.

### Reform of Poor-law Infirmaries.

There was a very strong case for taking special action with regard to Poor-law infirmaries. There could be such a co-ordination of activities and of capacities in regard to the care and cure of the sick poor as would set the whole system in order and prevent a large waste of public money, such as was inevitable now unless the Poor-law infirmaries were put in their proper position as part of the general system of medical relief. If the law were amended the cases of paupers could be concentrated—if the Poor Law still continued its nomenclature—in a certain number of infirmaries, and the others could be made available for the general purposes of treatment and relief.

The Earl of Onslow, Parliamentary Secretary to the Ministry of Health and Chairman of the new Central Hospitals Commission, explained the steps that the Ministry had taken to bring into effect the findings of Lord Cave's Committee. The question of building extensions would go before the local committees it was proposed to set up, and Lord Burnham's point as to infirmary accommodation being used to relieve that of voluntary hospitals would go before these committees and would be reported upon by them.

### The Position of the Teaching Hospitals.

Lord Dawson considered that the teaching hospitals should be put in a different schedule from the general hospitals. If anything interfered with the efficiency of the hospitals with medical schools the whole nation would suffer. Further, the question of the Poor-law infirmaries was an urgent one. The whole tendency of modern life was to increase the need for institutional treatment. The first reason was scientific: the diagnosis of disease was no longer the work of a single individual. It was done by co-operation, and that required equipment and organisation. Another reason was economical; complexity of the investigation of illness was increased, and that augmented the cost. The only solution of the efficient investigation of disease was an increased provision of institutional or mass treatment. The third reason was social, as it was becoming more difficult to deal with illness in the home, and this could be helped by the voluntary system being assisted, under certain conditions, by extended use of the Poor-law infirmaries.

